# Changes in Histone H3 Acetylation on Lysine 9 Accompany Aβ 1-40 Overexpression in an Alzheimer’s Disease Yeast Model

**DOI:** 10.17912/micropub.biology.000492

**Published:** 2021-11-02

**Authors:** Muna M. Hugais, Samantha N. Cobos, Seth A. Bennett, Jailene Paredes, Genevieve Foran, Mariana P. Torrente

**Affiliations:** 1 Department of Chemistry, Brooklyn College, Brooklyn, NY 11210; 2 PhD. Program in Chemistry, City University of New York - The Graduate Center, New York, NY, USA 10016; 3 PhD. Program in Biochemistry, City University of New York - The Graduate Center, New York, NY, USA 10016; 4 Ossining High School, Ossining, NY, USA 10562; 5 PhD. Programs in Chemistry, Biochemistry, and Biology, City University of New York - The Graduate Center, New York, NY, USA 10016

## Abstract

Alzheimer’s Disease (AD), the most common type of dementia, is a neurodegenerative disease characterized by plaques of amyloid-beta (Aβ) peptides found in the cerebral cortex of the brain. The pathological mechanism by which Aβ aggregation leads to neurodegeneration remains unknown. Interestingly, genetic mutations do not explain most AD cases suggesting that other mechanisms are at play. Epigenetic mechanisms, such as histone post-translational modifications (PTMs), may provide insight into the development of AD. Here, we exploit a yeast Aβ overexpression model to map out the histone PTM landscape associated with AD. We find a modest decrease in the acetylation levels on lysine 9 of histone H3 in the context of Aβ 1-40 overexpression. This change is accompanied by a decrease in RNA levels. Our results support a potential role for H3K9ac in AD pathology and allude to the role of epigenetics in AD and other neurodegenerative diseases.

**Figure 1. Aβ 1-40 overexpression in yeast is accompanied by changes in H3K9ac levels:**
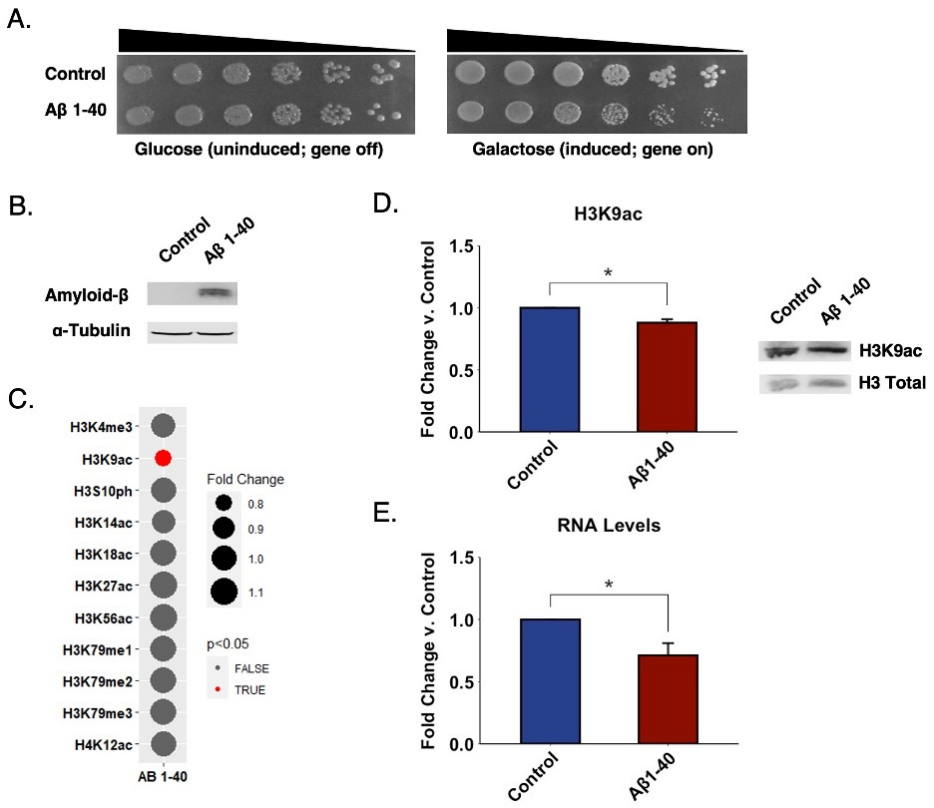
(A) Strains overexpressing Aβ 1-40 display mild growth suppression. Serial dilution growth assays for strains overexpressing Aβ 1-40 or a vector control grown in glucose or galactose-supplemented selective solid media. Black triangles indicate the relative concentrations of cells in the corresponding spots. (B) Expression of Aβ 1-40 verified by Western blotting. (C) Histone PTM levels are mostly unaffected by Aβ 1-40 overexpression in yeast. The bubble size represents the degree of change in the relative abundance of a histone modification. A smaller bubble represents a decrease in the levels of the modification, while a larger bubble represents an increase. The color scale depicts p-values derived from statistical analysis of Western blotting experiments. Red indicates changes with a p-value below 0.05, while gray indicates a p-value above 0.05. p-values were calculated using a two-tailed t test with Welch’s modification. (D) Acetylation on lysine 9 of Histone H3 is modestly decreased in the context of Aβ 1-40 overexpression. Graph displays the mean fold change in modification levels for Aβ 1-40 compared to vector control. Representative immunoblot shows the levels of lysine acetylation in Aβ 1-40 overexpression. Error bars indicate +SD (n=3). (*) p < 0.05. (E) Total RNA levels are decreased in Aβ 1-40 compared to vector control. Graph displays the mean fold change in total RNA levels for Aβ 1-40. Error bars indicate +SD (n=3). (*) p < 0.05.

## Description

Alzheimer’s Disease (AD) is a progressive and incurable neurodegenerative disease. The disease results in the gradual degeneration and eventual death of neurons causing complications with movement and mental function (Gao and Hong 2008). AD is characterized by plaques of amyloid-beta (Aβ) peptides in the cerebral cortex of the brain. Aβ peptides range from 37 to 49 amino acid residues in length (Chen *et al.* 2017). Aβ 1-40 and Aβ 1-42 are the most common species in the cerebral cortex of AD patient samples, with Aβ 1-40 being the most abundant (Seynnaeve *et al.* 2018; Spies *et al.* 2010). Aβ accumulation and aggregation are thought to lead to a series of neurodegenerative events (Novo *et al.* 2018). However, the factors triggering the aggregation of Aβ proteins have not been completely characterized. Furthermore, exactly how Aβ aggregation leads to neurodegeneration remains unclear (Wolfe and Cyr 2011).

The role of epigenetic mechanisms in the development of AD has become a subject of intense investigation. Epigenetics invokes changes in phenotype resulting from changes on chromatin structure and accessibility occurring without changes in the underlying DNA sequence (Esposito and Sherr 2019). Epigenetic mechanisms, such as histone post-translational modifications (PTMs), may provide insight into the development of AD. We hypothesize that the toxic effect of Aβ aggregation might be related to its association with altered histone marks. In fact, AD animal models and post-mortem brain samples from patients exhibiting a decline in gene expression might be mediated via histone PTMs (Esposito and Sherr 2019).

*Saccharomyces cerevisiae* models have become valuable tools in neurodegenerative disease research due to the genetic and environmental manipulations possible (Seynnaeve *et al.* 2018). Furthermore, upon expression of disease-related human proteins, yeast recapitulate many of the features of neurodegenerative diseases and display an easily observable disease phenotype (Bennett *et al.* 2019; Treusch *et al.* 2011). In addition, yeast models are affordable and time-efficient (Bennett *et al.* 2019; Seynnaeve *et al.* 2018). A powerful Aβ 1-40 yeast model has been previously developed by Treusch *et al.* where Aβ 1-40 is expressed and targeted to the secretory pathway. Aβ 1-40 peptides are targeted to the endoplasmic reticulum and transit throughout the secretory pathway, thus mimicking Aβ trafficking. Remarkably, a screen for Aβ toxicity modifiers using this model revealed a known risk factor for AD, genes associated with previously discovered AD risk factors as well as novel risk factors (Treusch *et al.* 2011). Here, we exploit this model to characterize the histone PTM landscapes associated with Aβ aggregation (Treusch *et al.* 2011).

We constructed the same yeast model, but in a different mating type for consistency with our experiments in other neurodegenerative disease proteinopathies (Chen *et al.* 2018). *S. cerevisiae* were transformed with either a control vector or a vector encoding for Aβ 1-40 and grown on galactose (inducing) and glucose (non-inducing) supplemented media. In agreement with previous work (Treusch *et al.* 2011), expression of Aβ 1-40 modestly impaired cell growth (**Figure 1A**). We verified expression of the Aβ peptide by Western blotting (**Figure 1B**).

We analyzed the histone PTM landscape associated with the overexpression of Aβ 1-40 by way of immunoblotting. We did not enrich for any particular genomic regions, but rather looked at global modification changes throughout the whole genome. We characterized various histone methylation, acetylation, and phosphorylation sites. We focused on histone H3 and H4 as these are most abundantly modified. We also focused on modifications conserved between yeast and human. In contrast to our findings in other neurodegenerative disease yeast models (Chen *et al.* 2018), we did not detect any significant changes in acetylation levels on lysine 14, 18, 27, or 56 of histone H3 or on lysine 12 of histone H4 (**Figure 1C**). Moreover, we did not detect any significant changes in the tri-methylation levels on H3K4 or in mono-, di-, or tri-methylation levels on H3K79 or in H3S10ph levels (**Figure 1C**). However, we discovered a modest, but highly reproducible decrease in the levels of H3K9ac in the context of Aβ 1-40 overexpression (**Figure 1D**). The magnitude of this change parallels the extent of growth suppression elicited by Aβ 1-40 (**Figure 1A**). Furthermore, these changes detected genome-wide are likely a significant underestimate of the potential greater difference detected at specific chromatin loci. Interestingly, decreases in H3K9ac were found in transgenic AD mouse models (Currais *et al.* 2019). Furthermore, treatment with HDAC inhibitors in mice reverses the decrease in histone acetylation levels leading to improved memory (Lu *et al.* 2015). Very recently, increases in H3K9ac have been linked to AD in post-mortem human brains and fly models (Nativio *et al.* 2020). Despite the differences in direction of change, we interpret the overlap between various model systems and patient tissues to point a true role for H3K9ac and its associated ‘writers’, ‘erasers’, and other binding partners at various stages of AD pathology.

Epigenetic factors regulate gene expression. Histone deacetylation results in a condensed chromatin structure leading to decreased gene expression (Shahid *et al.* 2021). To explore if the modest changes in H3K9ac observed are sufficient to impact gene expression, we chose a straightforward approach in which we extracted and quantitated total RNA levels in Aβ 1-40 yeast and compared it to control. Increases in total RNA levels suggest an up-regulation in gene expression, while decreases in RNA levels suggest a down-regulation in gene expression. We find a ~30 percent decrease in the RNA levels in the Aβ 1-40 strain compared to control (**Figure 1E**). Notably, we observed this effect despite protein overexpression. Therefore, we establish that decreases in H3K9ac are accompanied by decreases in global RNA levels.

In summary, we observe a modest, but highly reproducible decrease in the acetylation on lysine 9 of histone H3 in the context of Aβ 1-40 overexpression in *S. cerevisiae*. Furthermore, we observe a global decrease in RNA levels in the context of Aβ 1-40 suggesting decreased gene transcription. Our findings offer additional evidence supporting a role for H3K9ac in AD pathology and allude to novel diagnostic and therapeutic epigenetic tools for AD and other neurodegenerative diseases.

## Methods

**Yeast Transformations, Serial Dilution Growth Assays, and Protein Overexpression.** Yeast were transformed following standard procedure using poly(ethylene glycol) and lithium acetate (Gietz and Schiestl 2007). For growth assays, yeast were grown to saturation overnight at 30°C with shaking in synthetic dropout medium containing raffinose. Cultures were diluted 2-fold, serially diluted 5-fold, then spotted in duplicate onto plates containing synthetic dropout medium supplemented with glucose or galactose. Plates were incubated at 30°C for 2-3 days. Protein overexpression was induced in synthetic dropout medium supplemented with galactose for 8 hours at 30°C. After induction, cultures were normalized to an optical density of 0.6-0.8. 10 mL aliquots of cells were then harvested and frozen at -80°C.

**Western Blotting.** Western blotting analyses were performed as previously described (Bennett *et al.* 2019). Frozen yeast cell pellets were thawed and treated with 0.2 M NaOH for 10 minutes on ice, pelleted again, and resuspended in 100 µL of 1X SDS sample buffer and boiled at 95°C for 10 minutes. Cell lysates were separated via SDS-PAGE using 18% polyacrylamide gels and then transferred to a PVDF membrane (EMD Millipore). Membranes were blocked using LI-COR blocking buffer (LI-COR Biosciences, Lincoln, NE) for 1 hour at room temperature. Primary antibody incubations were performed at 4°C overnight. An anti-β-amyloid mouse antibody was used to verify expression of Aβ peptides and histone modification-specific antibodies were used for detection of histone PTMs. Blots were then processed using donkey anti-mouse (1:20,000 dilution factor) and donkey anti-rabbit (1:20,000 dilution factor) secondary antibodies and imaged on an Odyssey FC Imaging System (LI-COR Biosciences). All experiments were performed a minimum of three times with independent cell samples.

**RNA Purification.** Induced yeast aliquots were thawed and treated with 100 units of Zymolyase-20T (Nacalai USA, San Diego, CA; Cat# 07663-91) for 30 minutes at 30°C. RNA was isolated using the RNeasy Mini Kit from Qiagen (Germantown, MD; Cat# 74104) according to the manufacturer’s instructions. Total RNA levels were measured using a NanoDrop Lite spectrophotometer (Thermo Fisher Scientific, Waltham, MA). All experiments were repeated a minimum of three times with independent cell samples.

**Data and Statistical Analysis.** ImageJ Studio Software (LI-COR Biosciences) was used for densitometric analysis of Western blots. Individual histone modifications were quantitated by blot image analysis, normalized to the loading control, and compared with the control sample to obtain fold change measurements. The RNA concentration of the Aβ 1-40 sample was normalized against the control sample to obtain fold change measurements. Fold change values were then used for statistical analysis, which were performed in R 4.0.4 using the built-in stats package (R-Project, Vienna, Austria). Error bars on the bar graphs were used to represent standard deviation (SD). Welch’s T-test with p=0.05 as the cutoff for significance was used to determine any significant differences between sample groups (ccdB vs. Aβ 1-40).

## Reagents

**Table:** 

**Strain**	**Genotype**	**Reference**
W303a	*MATa*, *can1*–*100*, *his3*–*11*,*15*, *leu2*,*3*,*112*, *trp1*–*1*, *ura3*–*1*, *ade2*–*1*	(Sanchez and Lindquist 1990)
**Vector**	**Plasmid**	**Gifted from**
Control	pAG305GAL-ccdB	Susan Lindquist (Addgene, Cambridge MA)
Aβ 1-40	pAG305GAL- Aβ1-40	James Shorter (University of Pennsylvania)
**Antibody**	**Description**	**Company, Cat#**
α-tubulin	Rabbit monoclonal	Abcam, ab184966
β-amyloid	Mouse monoclonal	BioLegend, 803001
H3 Total	Mouse monoclonal	Abcam, ab24834
H3K4me3	Rabbit polyclonal	Abcam, ab8580
H3K9ac	Rabbit polyclonal	Abcam, ab10812
H3S10ph	Rabbit polyclonal	Abcam, ab5176
H3K14ac	Rabbit polyclonal	Millipore, 07-353
H3K18ac	Rabbit polyclonal	Abcam, ab1191
H3K27ac	Rabbit monoclonal	Abcam, ab45173 (Discontinued)
H3K56ac	Rabbit polyclonal	Active Motif, 39281
H3K79me1	Rabbit polyclonal	Millipore, ABE213
H3K79me2	Rabbit polyclonal	Abcam, ab3594
H3K79me3	Rabbit polyclonal	Abcam, ab2621
H4K12ac	Rabbit polyclonal	Abcam, ab46983
donkey anti-mouse	Mouse IgG	LI-COR Biosciences, 926-32212
donkey anti-rabbit	Rabbit IgG	LI-COR Biosciences, 926-68073
